# ﻿A new species of *Schlegelia* (Schlegeliaceae) from wet montane forest of Colombia and a key for the species of the genus

**DOI:** 10.3897/phytokeys.230.107398

**Published:** 2023-08-11

**Authors:** Gerardo A. Aymard Corredor, M. Alejandra Jaramillo

**Affiliations:** 1 UNELLEZ-Guanare, Programa de Ciencias del Agro y el Mar, Herbario Universitario (PORT), Mesa de Cavacas, estado Portuguesa 3350, Venezuela Herbario Universitario (PORT) Mesa de Cavacas Venezuela; 2 Compensation International Progress S.A. C.I. Progress-Greenlife, Bogotá, D.C., Colombia Compensation International Progress S.A. C.I. Progress–Greenlife Bogotá Colombia; 3 Grupo Diversitas, Facultad de Ciencias Básicas y Aplicadas, Universidad Militar Nueva Granada, km 2 vía Cajicá-Zipaquirá, Cundinamarca, Colombia Universidad Militar Nueva Granada Cajicá Colombia

**Keywords:** Climbing plants, Flora of Colombia, Lamiales, lianas, montane wet forests, Serranía de Las Quinchas, Virolín

## Abstract

In this paper we describe and illustrate *Schlegelialongirachis* a new species from montane forest remnants (1200--1900 m) in the Western slope of the Eastern Cordillera of Colombia (“Serranía de Las Quinchas” and Virolín county) in the Departments of Boyacá and Santander. A root-climbing liana, the new species is contrasted to *S.fuscata*, *S.monachinoi* and *S.parviflora*, the three most morphologically similar species of *Schlegelia*. This new species is differentiated from its putative close relatives by vegetative (texture, colour, pubescence and shape in leaves, bracts, bracteoles pedicel, calyx and corolla), inflorescences as well as floral characters (staminode absent). We provide an updated key to 24 known species of *Schlegelia*. For the identification key, *S.fuscata* and *S.roseiflora* are regarded here as different from *S.parviflora*. *S.urbaniana* is considered a synonym of *S.axillaris*, whereas *S.fastigiata* is separated from *S.sulphurea* as a recognizable species. *Schlegelia* has its center of distribution in Colombia, where 17 of the species are known to occur.

## Abstract (es)

En este artículo se describe e ilustra *Schlegelialongirachis* una nueva especie de los remanentes de bosques montanos húmedos (1200--1900 m) localizados en la vertiente occidental de la Cordillera Oriental de Colombia, en la Serranía de Las Quinchas y corregimiento de Virolín, en los departamentos de Boyacá y Santander (respectivamente). Esta nueva especie es una liana trepadora por raíces, la cual comparte varias similaridades con *S.fuscata*, *S.monachinoi* y *S.parviflora*. Sin embargo, difiere de estas especies en la textura, colores, pubescencia, forma de las hojas, brácteas, bractéolas, pedicelo, cáliz y corola, en el tipo de inflorescencia y por la ausencia del estaminodio. Se presenta una clave actualizada para diferenciar las especies del género *Schlegelia*. Para la clave, en un contexto geográfico y taxonómico, *S.fuscata* y *S.roseiflora* son tratadas como especies diferentes de *S.parviflora*. Por otra parte, *S.urbaniana* es considerada un sinónimo de *S.axillaris* y *S.fastigiata* es separada de *S.sulphurea*, y es reconocida como una especie válida. La presente contribución incrementa a 24 las especies de *Schlegelia*, 17 de éstas se conocen en Colombia, el país con la mayor diversidad del género.

## ﻿Introduction

*Schlegelia* comprises 24 species (including the new species described herein), as presently circumscribed in the key provided here. The genus occurs from the states of Chiapas, Oaxaca and Veracruz in Mexico (i.e., *S.nicaraguensis* Standl.; *sensu*Villaseñor Ríos 2016), the Antilles (i.e., *S.parasitica* (Sw.) Miers ex Griseb.), Central America, the Chocó Region, and Northern South America from the Guayana Shield to the Amazonia of Brazil, Colombia, Peru and Venezuela; at elevations from sea level to 2100 m (Gentry 1973, 1977, 1982a, 1982b, 1997, 2001, 2009). The genus is recognized by its conspicuous climbing habit, which consists of lianas climbing by adventitious roots, without tendrils (Gentry 1973, 1980). Its leaves are simple, with axillary pseudostipules (prophylls). The inflorescences are axillary racemes or terminal thyrses. Calyces are cupular or irregularly lobed. Corollas are tubular, tubular-campanulate infundibuliform-campanulate or hypocrateriform-campanulate. Petals are white, pink, red, yellow, or purple. Ovaries have an incompletely bilocular placenta. The fruit is a globose berry, up to 5 cm diam., with a persistent calyx (Gentry 1980, 2009).

*Schlegelia* was described from a collection made by Hendrick C. Focke, a Dutch Guianan lawyer, botanist, and ethnologist, who made numerous plant collections in Suriname between 1835–1850 (Pulle 1906). He sent his collections to Freidrich A. W. Miquel, who described the genus along with *S.lilacina* Miquel [= S.violacea (Aubl.) Griseb.; Miquel, 1844]. From its inception the relationships of *Schlegelia* were not clear; Miquel described the genus under the tribe Crescentiaeae as conceived by Endlicher (1839). Crescentieae was considered part of Gesneriaceae by Endlicher and consequently Miquel. However, Don, De Candolle, Martius and Fenzl considered Crescentieae part of Bignoniaceae (Miquel 1844).

*Schlegelia*, currently belongs into its own family, the SchlegeliaceaeReveal (1995: 74–75), a Neotropical family that includes four genera, two of them monotypic: *Exarata* Gentry (*E.chocoensis* A. H. Gentry), from the Chocó Region, and *Synapsis* Griseb. (*S.ilicifolia* Griseb.) from Cuba; and two relatively larger genera *Gibsoniothamnus* L.O.Williams (ca. 10 species) distributed in Mesoamerica and the Antilles, and *Schlegelia* Miq. (1844: 785). Before Schlegeliaceae was considered as a formal family by Reveal (1995), A. H. Gentry had proposed a new tribe Schlegelieae Gentry of the Bignoniaceae (Gentry 1980). The tribe was suggested as it was difficult to place these genera within Bignoniaceae or Scrophulariaceae (Gentry 1980; Armstrong 1985). Phylogenetic analyses confirmed Schlegeliaceae as monophyletic and distinct from Bignoniaceae and Scrophulariaceae (Spangler and Olmstead 1999; Olmstead et al. 2009). The most recent phylogenetic reconstruction based on chloroplast and nuclear genes place Schlegeliaceae (a) sister to Martyniaceae and Thomandersiaceae (BS<90, Liu et al. 2020); (b) sister to a clade including Pedaliaceae, Lentibulariaceae, Acanthaceae, Bignoniaceae and Verbenaceae (BS=98, 80 cp genes, Fonseca 2021); or (c) sister to Bignoniaceae and Verbenaceae (BS=65, 410 nuclear genes, Fonseca 2021). The relationship of Schlegeliaceae to other families of Lamiales is still not well understood; a better sampling of the family within molecular phylogenetic analyses should shed some light on the placement of Schlegeliaceae within this diverse order.

No comprehensive monograph of *Schlegelia* has been completed to date, although the genus has been treated largely as part of Bignoniaceae for Flora of Panama (Gentry 1973), Flora of Ecuador (Gentry 1977), Flora de Venezuela (Gentry 1982a), Flora de Veracruz (Gentry 1982b), Flora of the Venezuelan Guayana (Gentry 1997), Flora of Costa Rica (Burger and Barringer 2000), Flora de Nicaragua (Gentry 2001), Flora de Colombia (Gentry 2009) and Manual de Plantas de Costa Rica (Morales 2015). In addition, the genus has been treated in: Checklist of the plants of the Guiana Shield (Funk et al. 2007), Catálogo de las plantas vasculares nativas de México (Villaseñor Ríos 2016), Catalogue of seed plants of the West Indies (Acevedo-Rodriguez and Strong 2023), and Catálogo de plantas y líquenes de Colombia (Gradstein 2016).

The present work describes and illustrates a new species of *Schlegelia*, found in an isolated population located in highly fragmented montane forest. This new species was detected during the academic fieldwork conducted by “Herbario de la Universidad Militar Nueva Granada” (UMNG-H). Currently, the distribution of this new species is known only from “Serranía de Las Quinchas” and “Virolín” region, in Municipalities of Otanche and Charalá, Boyacá and Santander departments. Further botanical explorations of the area and the nearby municipalities are expected to uncover additional populations of this species as they share similar habitats. The present contribution increases to 24 the number of *Schlegelia* species, 17 of them known from Colombia, the country with the highest diversity of the genus.

## ﻿Materials and methods

We examined 120 herbarium specimens of *Schlegelia* from South America deposited at “Herbario de la Universidad Nacional de Colombia” (COL). In addition, all type specimens, as well as general collections, hosted by virtual herbaria, were consulted, including those maintained by the Field Museum (F; http://emuweb.fieldmuseum.org/botany/taxonomic.php), Instituto Nacional de Pesquisas da Amazônia (INPA; http://inct.florabrasil.net/en/), JSTOR Global Plants (http://plants.jstor.org), Museum of Natural History, Paris (P; http://www.mnhn.fr), Reflora Virtual Herbarium (http://reflora.jbrj.gov.br/reflora/), speciesLink (https://specieslink.net/), Smithsonian Institution (US; https://collections.si.edu/search/), Universidad de Antioquia, Colombia (HUA; http://www2.udea.edu.co/herbario/paginas/consultas/consultarEjemplares.iface), Universidad Nacional Autónoma de México (MEXU; https://datosabiertos.unam.mx/biodiversidad/), and the National Herbarium of The Netherlands (U; https://www.nationaalherbarium.nl/). The herbarium codes after Thiers (2019).

This publication is based on morphological assessments of herbaria collections. The description of the new species is based on field observations (flower and fruit material was preserved in ethanol) as well as on herbaria specimens. The flowers from herbaria specimens were rehydrated for three days before measuring using a 1:1 combination of glycerin and 0.9 NaCl solution.

Plants of the World (POWO, https://powo.science.kew.org) and taxonomic literature on *Schlegelia* were consulted to assemble the species key; in particular, Bignoniaceae for Flora of Panama (Gentry 1973), Flora of Ecuador (Gentry 1977), Flora of Venezuela (Gentry 1982a), Flora of Venezuelan Guayana (Gentry 1997) and Flora of Colombia (Gentry 2009). The Catálogo de plantas y líquenes de Colombia (Gradstein 2016) was also reviewed. Additionally, the International Plant Names Index (https://www.ipni.org/), the online botany collections of the Smithsonian National Museum of Natural History (https://naturalhistory.si.edu/research/botany), and Tropicos (http://legacy.tropicos.org/Home.aspx) were consulted to update the current nomenclature and geographical information. Terminology for vegetative characters, inflorescences, flowers, and fruit morphology follow Gentry (1977, 2009) and Font-Quer (2001).

To determine the conservation status (according to IUCN categories and criteria; IUCN Standards and Petitions Committee 2022), the extent of occurrence (EOO) and area of occupancy (AOO) were calculated using the supporting Red List threat assessments with GeoCAT (Bachman et al. 2011), which is continually updated (https://geocat.kew.org/). The EOO is defined by the IUCN Standards and Petitions Committee (2022) as the minimum convex polygon encompassing all known occurrences of a species. In addition, AOO is the area within the EOO, which is comprised of 2 × 2 km grid cells containing known occurrences records.

## ﻿Taxonomic treatment

### ﻿ Schlegelia longirachis

Taxon classificationPlantaeLamialesSchlegeliaceae

Aymard & M.A.Jaram. sp. nov.

5EF45051-5A07-5B83-9FF2-E9882BBE016E

urn:lsid:ipni.org:names:77325154-1

#### Type.

**Figure 1.**
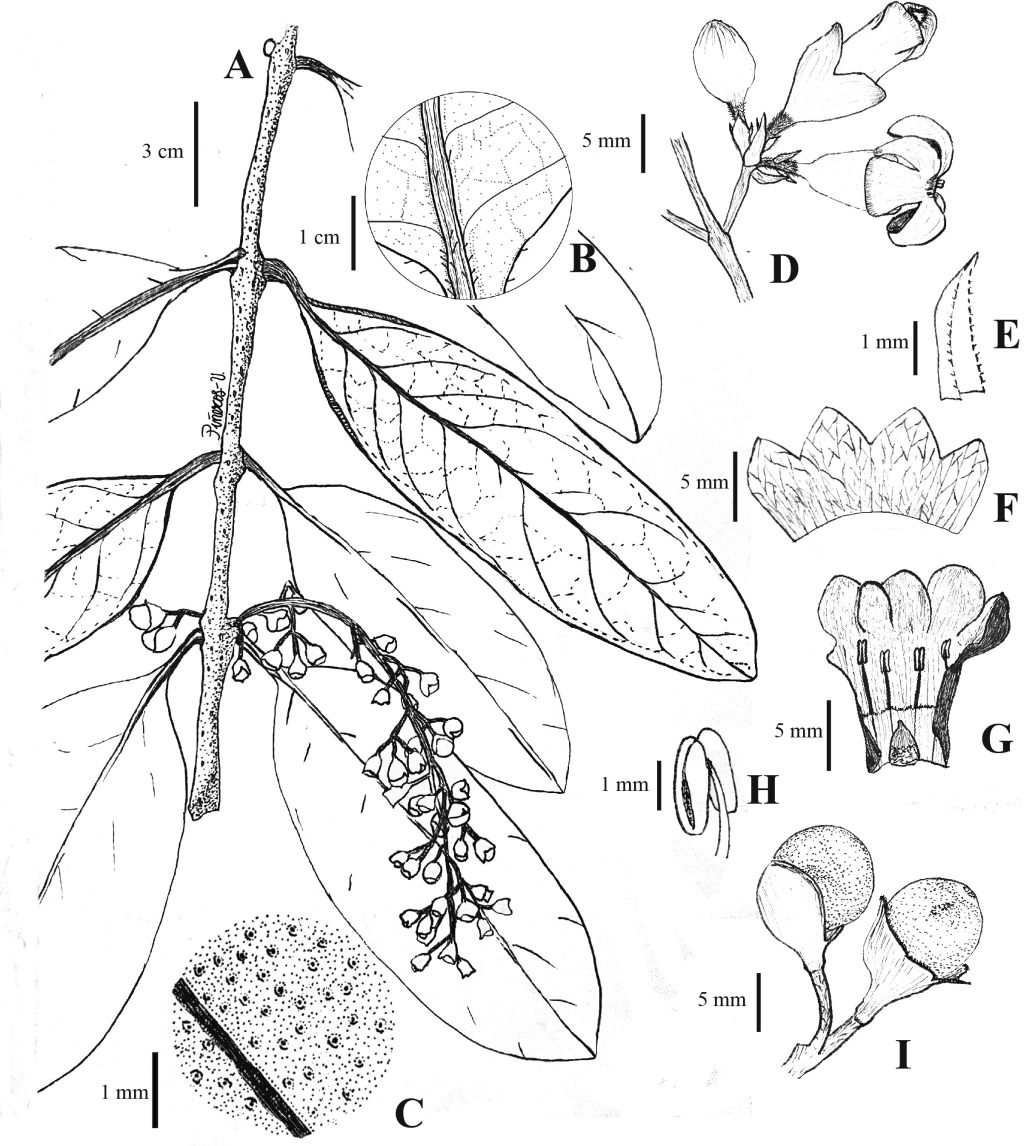
*Schlegelialongirachis***A** a flowering branch **B** detail of the leaf abaxial surface **C** detail of the leaf adaxial surface showing the black punctations **D** inflorescence showing the bract and bracteoles **E** bract **F** calyx extended **G** corolla and adnate stamens extended **H** detail of a stamen **I** fruits. Illustration by Paola Piñeros.

**Figure 2.**
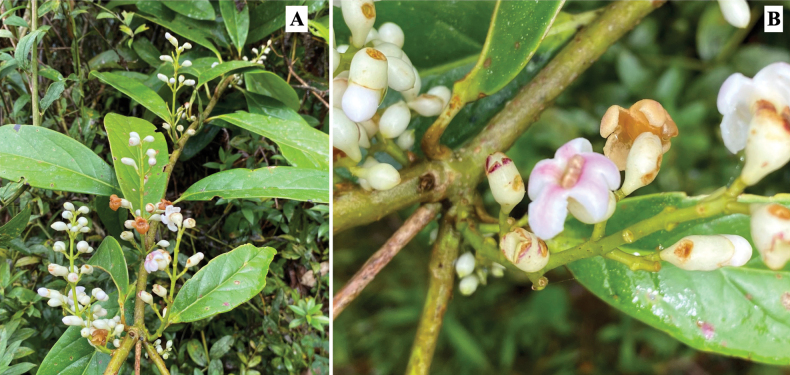
*Schlegelialongirachis***A** a flowering branch **B** detail of the inflorescence. Photos by Andrés Majín.

Colombia. Boyacá. Municipio Otanche. Serranía de Las Quinchas, sector la Y, Finca Lote Terreno, 5°41'42.6"N, 74°19'37.5"W, 1200 m, 26 Oct 2022 (fl, fr). *M. Alejandra Jaramillo*, *Andrés F. Majin-Ladino*, *Lucindo Galvis & estudiantes de Taxonomía vegetal 2022-1.* (Holotype: COL!; Isotypes: UMNG-H!, HUA!). Figs 1, 2.

*Schlegelialongirachis* resembles *S.monachinoi*, but can be differentiated from this species by the longer internodes, 4–8cm long in *S.longirachis*, vs. 1.5–4.5cm in *S.monachinoi*. The leaf blades densely black punctuated on the adaxial surface, vs. sparsely punctuated towards the base of the blade on both surfaces in *S.monachinoi*. The inflorescences are longer 4–18 cm long in *S.longirachis*, vs. 3–11 cm in *S.monachinoi*. Bracts are oblong vs. lanceolate-triangular in *S.monachinoi*.

#### Description.

***Root-climbing liana*** internodes 4–8 cm long, ca. 3cm in diameter, pale brown when dry, branches sparsely lenticelate. ***Leaves*** simple, opposite; petioles 12–20 mm long, glabrous; leaf blade lanceolate, lanceolate-elliptic, rarely oblanceolate, 4–22 × (3.2) 4.5–9 cm; coriaceous, densely black punctuated on the adaxial surface (Fig. 1C), glabrescent or with simple trichomes located near base and in the midrib on the abaxial surface (Fig. 1B), base obtuse-rounded or cuneate, apex rounded or acute, margins entire, black-brown upon drying; venation brochidodromous, midrib prominent on the abaxial surface, 6–7 pairs of secondary veins, the tertiary veins conspicuously reticulate on the abaxial surface. ***Inflorescence*** axillary, narrowly thyrsic with dichasial partial inflorescences; rachis puberulous to sparsely adpressed pubescent (5–12 cm long in flower, 12–18 cm long in fruit); flowers 14–20, produced in long-peduncled, 2–3-flowered dichasia along the rachis, each flower subtended by a bract and 2 bracteoles (Fig. 1D), bracts 2–3 mm long, oblong, glabrous, ciliate at the margins; bracteoles ca. 1 mm long, triangular, ciliate at the margins; pedicels 3.5–4.5 mm in flower, 7.5–8.5 in fruit, adpressed pilose. ***Calyx*** cupular ca. 6 × 5 mm, bilabiate fused, 4-lobed, lobes oblong, 2–2.5 (3.2) mm long, apex rounded-acute, white, sparsely puberulent and with white disk-shape glands on the outer surface (visible in dry collections), glabrous and reticulate veined inside. ***Corolla*** campanulate-hypocrateriform with 5 reflexed lobes, white, deep pink at the throat (Fig. 2B); tube 7–8 mm long, ca. 4 mm wide in the mouth; lobes 3–4 × 3 mm, glabrous inside, minutely puberulous outside. ***Stamens*** didynamous (Fig. 1H), subexserted, filament 3–4 mm long, pilose at the base, inserted at ca. 4 mm from base of corolla; anthers ca. 1.5 mm long, oblong, glabrous; staminode absent. ***Pistil*** with conical ovary, ca. 1.5 × 1.5 mm, glabrous; nectariferous disk fused and not clearly differentiated from the ovary base. ***Fruit*** a berry, 6.6–8 mm in diam., spherical, drying black, glabrous with conspicuous papillae, covered to the middle by a persistent calyx (Fig. 1I, 2B). **Seeds** not seen.

#### Phenology.

Collected in flower in March, and in flower and fruit in October.

#### Etymology.

The specific epithet refers to the long rachis of the inflorescences that is present in this new species. The long rachis of *S.longirachis* displays the flowers away from the foliage, a characteristic that may have some bearing on the pollination strategy of the species.

#### Distribution and habitat.

The species is known to occur in montane forest remnants between 1200 and 1900 m. In the type locality, *S.longirachis* grows in forest consisting of medium to tall trees.

#### Conservation status.

This species is known only from the type and two additional localities; however, it is reported here as a very rare species. It should be regarded as Endangered (EN) due to the low number of known localities, its estimated Area of Occupancy (AOO) of 12,000 km^2^, and its estimated Extent of Occurrence (EOO) of 755,768 km^2^ (IUCN Standards and Petitions Committee 2022). Additionally, the conservation of these forests is at risk due the continuous deforestation and degradation of the “Serranía de Las Quinchas” and their surrounding areas on middle Magdalena river, and the Virolín Region (Galindo-T. et al. 2003) especially in the years during the pre- and post-conflict period (peace agreement was signed in 2017). The expansion of deforestation, degradation and water pollution continues (Salgado et al. 2022), with significantly greater agricultural use, pasture, selective logging, illicit crops and mining (Restrepo et al. 2021). Although conservation status assessments can be made for species with such small numbers of collections (Rivers et al. 2011), it may be difficult to assess whether the appearance of rarity in a species is due to the lack of, or outdated, data, or to its actual rarity (Verspagen and Erkens 2022). Fortunately, the area where *S.longirachis* occurs is protected as part of the Regional Natural Park Serranía de las Quinchas (Stiles and Bohórquez 2000; Bohorquez-Osorio et al. 2020) and Fauna and Flora Santuary Guanentá-Alto Rio Fonce (Galindo-T. et al. 2003).

#### Additional specimens.

Colombia. Santander. Municipio de Charalá. Corregimiento de Virolín. Vereda El Reloj, Camino a Olival, aprox. 6°08'N, 73°20'W, 1680–1700 m, 03 Mar 1981, *S. Díaz Piedrahita 2273* (COL); Municipio de Charalá, Virolín, Vereda Palmar, 6°03'44.8"N, 73°12'50.4"W, 1894 m, 06 Oct 2009, *M. Blanco et al. 53* (COL).

#### Notes.

The species described here is morphologically similar to three taxa *S.fuscata* A. H. Gentry *S.monachinoi* Moldenke and *S.parviflora* as characterized in Table 1. However, it is most similar to *S.monachinoi* from the Andean wet forests in Colombia, Ecuador and Venezuela (Gentry 1977, 1982a, 2009). Both species have elongate, narrow axillary thyrses, the corolla and lobes lilac inside and fruit 0.5–2.5 cm in diam. *S.longirachis* differs from *S.monachinoi* in the characters presented in diagnosis. The new species can be distinguished with the key to the species presented below.

**Table 1.** Comparison of diagnostic morphological characters of *Schlegelialongirachis*, with morphologically similar species. ^1^In the *S.monachinoi* description, H. Moldenke mentioned that the bract is lanceolate, 2–5 mm long (Moldenke 1949). However, Gentry (2009) quoted that the bract is triangular, 1–2 mm long. Our examination of the type material deposited in COL confirmed the Gentry´s observation.

Character	*S.longirachis*	*S.fuscata*	*S.monachinoi*	*S.parviflora*
Leaves	3–9 cm wide, lanceolate, lanceolate-elliptic, rarely oblanceolate, coriaceous, with simples trichomes on the abaxial surface, black-brown when dry, base obtuse-rounded or cuneate	5–12 cm wide, widely-elliptic to elliptic, oblanceolate, rigid-coriaceous, with lepidote trichomes and disk-shape glands located near base of midrib on the abaxial surface, brown when dry, base truncate or widely-cuneate	5–11 cm wide, elliptic, oblanceolate, rarely narrowly ovate, rigid-coriaceous, with lepidote trichomes and disk-shape glands located near base of midrib on the abaxial surface, yellowish when dry, base acute	4.5–15 cm wide, obovate or elliptic-obovate, coriaceous, glabrescent or with lepidote trichomes and disk-shape glands located near base of midrib on the abaxial surface, olive green or brown when dry, base cuneate
Inflorescences	4–18 cm long, elongate, racemose to narrowly paniculate, puberulent or glabrous	1–(4)–5 cm long, shorter, racemose or narrowly subpaniculate, inconspicuous puberulent	4–15 cm long, elongate, racemose to narrowly paniculate, densely hirsute-puberulent (with lax trichomes)	2–5 cm long, shorter, contracted panicle, almost often fasciculate, glabrous to inconspicuous puberulent
Bract	2–3 mm long, oblong, sparsely puberulent outside, ciliate at the margins	2–5 mm long, lanceolate, glabrous on both sides, ciliate at the margins	1–2 mm long, triangular**^1^**, densely puberulous on both sides, short-puberulous along the margins	1–2 mm long, subulate, short-puberulous at least along the margins
Calyx	ca. 6 × ca. 5 mm, basally fused, 3–4-lobes, sparsely puberulent outside, brown when dried	6–7 × 5–6 mm, 3–5-lobed, inconspicuous lepidote, puberulent at the apex outside, black when dried	4–6 × 3--5 mm, 2–3-lobed, inconspicuous lepidote or subpuberulous at least in the base, yellowish-brown when dried	4–6 × 3–5 mm, 2–3-lobed, glabrescent or inconspicuous lepidote or subpuberulous at least in the base, brown when dried
Corolla	7–8 mm long, 4 mm wide in the mouth, campanulate-hypocrateriform, lilac inside, lobes 3–4 mm long, minutely (not-glandular) puberulous outside	10–11 mm long, 4 mm wide in the mount, tubular, lilac inside, lobes 3–4 mm long, glandular-lepidote to glandular puberulous inside	10–12 mm long, 5 cm wide in the mount, tubular, lilac inside, lobes ca. 5 mm long, glandular-lepidote to glandular puberulous inside	10–12 mm long, 5 cm wide in the mount, tubular, yellow inside, lobes 4–6 mm long, glandular-lepidote to glandular puberulous inside
Staminode	Absent	Present	Present	Present

^1^In the *S.monachinoi* description, H. Moldenke mentioned that the bract is lanceolate, 2–5 mm long (Moldenke 1949). However, Gentry (2009) quoted that the bract is triangular, 1–2 mm long. Our examination of the type material deposited in COL confirmed the Gentry´s observation.

### ﻿Key to the species of *Schlegelia*

Modified from Gentry (2009) species indicated with an asterisk (*) are endemic to Colombia.

Careful analysis of the literature and herbarium specimens led us to deem *Schlegeliafuscata* A. H. Gentry and *S.roseiflora* Ducke to be different from *S.parviflora* (Oerst.) Monach (see Table 1). *Schlegeliaurbaniana* K. Schum. ex Duss is considered a synonym of *S.axillaris* Griseb., whereas *S.fastigiata* Schery in separated from *S.sulphurea* Diels as a recognizable species.

**Table:** 

1	Inflorescences cauliflorous, ramiflorous	**2**
–	Inflorescences terminal or axillary	**10**
2	Corolla tubular-campanulate, > 3.5 cm long, ca. 1.1 cm wide at the mouth of the tube, purple or magenta, rarely white; lobes > 5 mm long; fruit ca. 4 cm diam.	**3**
–	Corolla tubular or narrowly tubular, 0.8–2.5 cm long, 0.2–0–4 cm wide at the mouth of the tube, white with apex pink, yellow, red or orange; lobes 1–4 mm long; fruit 1–1.5 cm diam.	**4**
3	Leaves strongly coriaceous, bullate, usually > 30 cm long; inflorescences a multifloral thyrse, densely contracted, subtended by a conspicuous fascicle of basal bracts	***S.dresslerii* A.H.Gentry (Panamá, Colombia, Ecuador)**
–	Leaves subcoriaceous or coriaceous, not bullate, usually < 11 cm long; inflorescences a pauciflorous thyrse; not subtended by basal bracts	***S.nicaraguensis* (México, Mesoamérica, Colombia)**
4	Pseudostipules present; corolla tube white (the lobes apex and calyx pink), pink or yellow; inflorescences a crowded (densely branched) or slightly contracted thyrse	**5**
–	Pseudostipules inconspicuous or absent; corolla (tube and lobes) red, red-orange or red-purple, calyx red or brown; inflorescence a paucifloral thyrse	**8**
5	Inflorescence a crowded, densely branched thyrse; corolla tube 1.8–2.5 cm long; ovary lepidote	**6**
–	Inflorescence a slightly or laxer contracted thyrse; corolla 0.8–1.2 cm long; ovary glabrous	**7**
6	Pseudostipules subulate; corolla tube yellow	***S.sulphurea* (Panamá, Colombia; Ecuador)**
–	Pseudostipules lanceolate; corolla tube white (the lobes apex and calyx pink)	***S.fastigiata* (Guatemala, Costa Rica, Panamá, Colombia, Ecuador)**
7	Pseudostipules subulate; leaves 10–25 × 7–20 cm, elliptic or obovate; corolla tube white (the lobes apex and calyx pink) or pink; 1–1.2 cm long	***S.macrophylla* Ducke (Brazil, Colombia, Perú)**
–	Pseudostipules lanceolate; leaves 7–11 × 2.5–5 cm, elliptic-oblong or obovate-oblong; corolla tube pink, ca. 0.8 cm long	***S.roseiflora* (Brazil, French Guiana, Perú)**
8	Leaves densely hirsute along primary and secondary veins on abaxial surface; primary and secondary veins impressed on the adaxial surface	***S.hirsuta* A.H.Gentry (Colombia)**
–	Leaves glabrous or lepidote on the abaxial surface, primary and secondary veins flat on the adaxial surface	**9**
9	Leaves chartaceous to subcoriaceous, elliptic to wide-elliptic, two times as long as wide, 15–26 cm long, the base auriculate with rolled up lobes; inflorescence thyrse with reduced partial inflorescences; calyx 5–7 mm long; corolla tube 2–2.5 cm long, red-purple	***S.spruceana* K. Schum. (Brazil, Colombia, Guyana, Venezuela)**
–	Leaves coriaceous, narrowly elliptic, more than two times longer than wide, 9–16 cm long, the base rounded or cuneate; inflorescences glomerulate, of several condensed thyrses; calyx 3–5(–6) mm long; corolla tube 1.8–2 cm long, red	***S.cauliflora* A.H.Gentry (Brazil, Colombia, Perú)**
10	Inflorescences terminal, 14–40 cm long	**11**
–	Inflorescences axillary, 0.5–21 cm long	**13**
11	Inflorescences with foliaceous bracts, 1–2.5 × 1–2 cm; a species endemic to the Choco Region	***S.darienensis* Sandwith (Colombia, Ecuador, very probably Panamá)**
–	Inflorescences with obsolete bracts, 1–2× ca. 1 mm; Amazonian and Guiana Shield species	**12**
12	Calyx subtruncate, 4–5 mm long; corolla tube ca. 2 mm wide; fruit 1–1.6 cm diam., 1/3 to 1/4 covered by a persistent, subtruncate calyx	***S.scandens* Sandwith (Brazil, Colombia, Perú, Suriname, Venezuela)**
–	Calyx irregularly 2–3-labiate, 5–9 mm long; corolla tube ca. 3 mm wide; fruit ca. 1 cm diam., with lower 2/3 covered by a persistent, distinctly toothed calyx	***S.violacea* (Brazil, Guianas, Venezuela)**
13	Fruits 3.5–5 cm in diam.	**14**
–	Fruit 0.5–2.5 cm in diam.	**15**
14	Leaves broadly obovate or rarely elliptic, coriaceous, apex rounded, base acute and decurrent on petiole, not lepidote; inflorescences 2.5–3.5 cm long, hispidulous; fruits 4.5–5 cm diam	***S.macrocarpa* Lundell (Guatemala)**
–	Leaves elliptic-obovate, chartaceous, or subcoriaceous, apex apiculate, base broadly cuneate; sparsely lepidote on both surfaces; inflorescences 1–1.2 cm long, puberulent; fruits 3.5–4 cm diam	***S.nicaraguensis* (México, Mesoamérica, Colombia)**
15	Leaves panduriform (fiddle shape), the base strongly auriculate	***S.pandurata* (Moldenke) A.H.Gentry (Colombia, Ecuador)**
–	Leaves elliptic, obovate, elliptic-obovate, wide-ovate, lanceolate, oblanceolate, oblong-ovate or oblong-elliptic, the base cuneate, rounded or abrupt subcordate, slightly or not auriculate	**16**
16	Corolla golden yellow, lobes 1–2 mm long; calyx toothed, lobes 2–2.5 mm	***S.aurea* Ducke (Brazil)**
–	Corolla white with pink tip, lilac, creamy or purple, lobes 3–6 mm long; calyx truncate, subtruncate or slightly toothed, lobes 0.5–1 mm long	**17**
17	Inflorescences a crowded, contracted thyrse, densely branched, the branchlets short and conspicuously jointed	***S.sulphurea* (Panamá, Colombia, Ecuador)**
–	Inflorescences lax thyrses or axillary thyrsic fascicles, 1–several flowered	**18**
18	Inflorescences fasciculate or very branched thyrses; corolla campanulate or infundibuliform-campanulate, 5– 6 mm wide toward the end of the tube	**19**
–	Inflorescences contracted or elongate thyrses, more or less fasciculate (*S.parviflora*) or a lax thyrse; corolla campanulate-hypocrateriform or tubular, 0.4–0.5 cm wide toward the end of the tube	**23**
19	Inflorescences a very short thyrse; corolla tube 0.6–0.8 cm long	**20**
–	Inflorescences fasciculate thyrses; corolla tube 1–3.5 cm long	**21**
20	Leaves 2.5–5 cm wide, elliptic-oblong or obovate-oblong; corolla tube pink, ca. 0.8 cm long	***S.roseiflora* (Brazil, French Guiana, Perú)**
–	Leaves 7–9 cm wide, widely obovate or widely elliptic; corolla tube white, 5–6 cm long	***S.axillaris* (Antilles)**
21	Leaves 4–7 cm long,, obovate; corolla 1.3–1.9 cm long	***S.brachyantha* Griseb. (Antilles, Colombia, Costa Rica, Panamá, Venezuela)**
–	Leaves 10–20 cm long, elliptic, oblong or elliptic-oblong; corolla 2.5–3.5 cm long	**22**
22	Leaves coriaceous; calyx tubular-campanulate, ca. 1 cm long, green; corolla ca. 3.5 cm long	***S.paraensis* Ducke (Brazil, Guianas, Venezuela)**
–	Leaves chartaceous; calyx campanulate, 0.4–0.5 cm long, violet; corolla 2.5–3 cm long	***S.parasitica* (Antilles)**
23	Young branches with conspicuous and dense, raised lenticels; base of leaves abruptly truncate or subcordate; petioles stout, 0.5–1.3 cm long; corolla 1.2–1.3 cm long, white with yellow throat	***S.chocoensis* A.H.Gentry (Colombia, Ecuador, very probable in Panamá)**
–	Young branches with inconspicuous or sparse lenticels; base of leaves rounded, cuneate or nearly so; petioles slender, 1–2.5 cm long; corolla ≤ 1.2 cm long, white or lilac or lavender, the throat lilac or lavender	**24**
24	Leaves 13–30 cm long; inflorescences a contracted thyrse, the main axis 1–(4)–5 cm long	**25**
–	Leaves 4–22 cm long; inflorescences thyrse or a lax thyrse, the main axis (4)–18 cm long	**26**
25	Inflorescences a slightly contracted thyrse; peduncle and pedicel stout and woody	***S.macrophylla* Ducke (Brazil, Colombia, Perú)**
–	Inflorescences a contracted thyrse, almost often fasciculate; peduncle and pedicel slender and herbaceous	***S.parviflora* (Mexico, Mesoamerica, Brazil, Colombia, Ecuador, French Guiana, Peru, Venezuela)**
26	Leaves widely-elliptic to elliptic or oblanceolate, brown when dry; inflorescences 1–(4)–5 cm long, narrowly thyrsic, calyx black when dried	***S.fuscata* A.H.Gentry (Nicaragua, Costa Rica, Panamá, Colombia, Ecuador, French Guiana, Venezuela)**
–	Leaves lanceolate, lanceolate-elliptic, elliptic, rarely narrowly ovate, or oblanceolate, black- brown or yellowish when dry; inflorescences 4–18 cm long, narrowly thyrsic; calyx brown to yellowish when dry	**27**
27	Leaves lanceolate, lanceolate-elliptic, coriaceous, glabrescent or with simple trichomes, densely punctuated on the adaxial surface, black-brown when dry; inflorescences rachis puberulent to sparsely pilose; bracts 2–5 mm long, oblong, ciliate along the margins; calyx sparsely puberulent on outer surface, brown when dry; staminode absent	*S.longirachis* * (Colombia)
–	Leaves elliptic, oblanceolate, rarely narrowly ovate, rigid-coriaceous, with lepidote trichomes and sparsely punctuated near base of midrib on both surfaces, yellowish when dry; inflorescences rachis densely hirsute-puberulent, bracts 1–2 mm long, triangular, short- puberulous along the margins; calyx lepidote or subpuberulous at least at the base, yellowish when dry; staminode present	***S.monachinoi* (Colombia, Ecuador, Venezuela)**

## Supplementary Material

XML Treatment for Schlegelia longirachis

## ﻿Additional information

### Author ORCIDs

Gerardo A. Aymard Corredor https://orcid.org/0000-0001-9405-0508

M. Alejandra Jaramillo https://orcid.org/0000-0002-6539-4149

### Data availability

All of the data that support the findings of this study are available in the main text.
